# Anopheles larval ecology and physicochemical characterization of larval habitats in Dire Dawa: an area colonized by Anopheles stephensi in Eastern Ethiopia

**DOI:** 10.21203/rs.3.rs-8442371/v1

**Published:** 2026-01-23

**Authors:** Ephrem Abiy, Teshome Degefa, Meshesha Balkew, Hailu Merga, Denekew Zewdu, Alamayo Ayala, Harrysone Atieli, Ming Chieh, Guofa Zhuo, Guiyan Yan, Delenasaw Yewhalaw

**Affiliations:** Jimma University; Jimma University; Former staff of Abt Global; Jimma University; Jimma University; Jimma University; University of California at Irvine; University of California at Irvine; University of California at Irvine; University of California at Irvine; Jimma University

**Keywords:** Physico-chemical characterization, mosquito larval habitat, Anopheles stephensi, larval density, Ethiopia

## Abstract

**Background:**

Understanding mosquito larval ecology is essential for planning and implementations of vector control strategies. The biotic and abiotic factors affect larval occurrence , density, survival and morphogenesis of mosquitoes. Artificial containers are very suitable larval habitats for some species of Anopheles and Aedes mosquitoes in urban and peri-urban settings. Therefore, this study we identified, mapped and characterized larval habitats, estimated larval density and indices larval habitats. In addition, we determined the species composition of *Anopheles* mosquitoes and species evenness in urban, peri-urban and rural areas of Dire Dawa city adminstration

**Methods:**

Larval habitats were surveyed and identified monthly for a period of 16 months from February 2023 to December 2024 in urban, peri-urban and rural areas of Dire Dawa. Mosquito larvae and pupae were collected and those larvae identified as *Anopheles* were fed on fish-food. Emerged adults were provided with 10% sucrose solution and kept under standard conditions in field insectary. Females *Anopheles* was identified morphologically and further species-specific PCR assay was employed to identify members of *An.gambiae* s.l. In addition, real time PCRassay was performed to identify *An.stephensi* and *An.arabiensis*. Water samples were taken from the larval habitats and the physico-chemical parameters were measured using HANNA Multi-parameter (H198194). Larval habitat diversity, larval abundance and distribution were assessed across the three ecological settings (urban, peri-urban and rural).

**Results:**

A total of 23, 526 larvae and 1,808 pupae of *Anopheles* mosquitoes were collected from 909 man-made(uncovered cemented cisterns (Brick), plastic sheets, steel drums, Tire tracks, Canal ditch, plastic tanker/Barrel) and natural habitats (River edges, ponds, animal hoof prints and swaps) in urban, peri-urban areas of Dire Dawa. The highest mean larval density (51 larvae per dip) of *Anopheles* mosquitoes was recorded from peri-urban sites in uncovered water tanker (brick) followed by urban site in brick (46 larvae per dip). *Anopheles* larvae were not found in steel drum and plastic barrels in rural sites.

A total of 2,934 adult *Anopheles* mosquitoes were emerged from immatures collected from all sites, of which 75% (2194/2934) were *An. stephensi*, and 22 % (636 / 2934) were *An.arabiensis*. The remaining 3.0% were *An. pharoensis*, *An. coustani*, *An .amharicus,* and *An. pretoriensis. Anopheles stephensi*, *An. arabiensis* and *An. amharicus* shared the same habitats across the three ecologies. Larval density was positively correlated with availability of brick making, proximity to houses, urban setting, presence of competitors /predators, vegetation cover, shade cover and substrate type. But larval presence was not correlated with presence/absence of intervention. Larva/pupa presence were positively correlated with pH (r=0.264, *p*=0.01) and water pressure (r=0.21, *p*<0.05).There was positive correlation among temperature, electrical conductivity (EC), total dissolved solids (TDS), salinity and dissolved oxygen (DO) and negative correlation among temperature with resistivity, pH with mvPH. Larval presence was positively correlated with water salinity and pH.

**Conclusion:**

*Anopheles stephensi* was the predominant species found in the study area, followed by *An.arabiensis, An.amharicu, An.pharoensis, An.coustani and An.pretoriensis*. Uncovered water tankers (Bricks) were the most prolific artificial habitats in urban and peri-urban sites followed by plastic sheets while natural habitats such as hoof prints and river basins were the most efficient habitat types in rural and urban settings, respectively. *Anopheles stephensi* was found in natural habitats of Butuji and legehare rivers from urban site, and in rural sites from man-made habitat of plastic sheet.

*Anopheles amharicus* larvae was found in plastic sheet, an artificial habitat common in urban, peri-urban and rural areas.

We report here the occurrence of *An.stephensi* in rural areas, breeding in natural habitats, and co-existing with *An. gambiae s.l* complex.

Availability of brick making, shorter distance from living houses being in urban ecology were directly correlated with larval density. Habitat abundance and positivity of uncovered water tankers (cemented cisterns or bricks) in urban and pre-urban sites could indicate for feasibility and proper implementation of larval source management.

## Introduction

*Anopheles* larval habitat characterization refers to the process of identifying and describing the environmental conditions where *Anopheles* mosquitoes lay their eggs and where their larvae develop [1]. These habitats are typically shallow bodies of water, such as ponds, swamps, streams, rice fields, and containers, which can vary in size and water chemistry. The characterization includes factors like water depth, vegetation, temperature, pH, salinity, and the presence of organic matter, as these conditions influence the survival and development of the larvae [2-4]. Understanding these habitats is crucial for malaria control, as targeting larval habitats with appropriate interventions, such as biological or chemical control methods, can help reduce mosquito populations and limit the transmission of malaria.

Each malaria vector has its own larval habitat preferences for oviposition that might be linked with their genetics make up or environmental parameters that imposed behavioral variation [5].

The main malaria vector *An.gambiae* complex prefers to breed in clean water of temporary rain pools, hoof prints, natural pockets of river, saline water (some species). The *An.funestus* groups breed in emergent vegetation and year round river sides, breeds in rice fields, and irrigation schemes. But the invasive *An.stephensi* breeds in artificial small containers, jars, bricks, plastic sheets, barrels, iron tankers, steel drums, used tire, ditches sometimes even in polluted water and fountains [6, 7].

Mosquito breeding habitats have specific biotic and abiotic factors that make them suitable to oviposition and support development of mosquito life cycle or metamorphosis [1, 8]. Studies conducted in Ethiopia on *Anopheles gambiae* s.l. showed that the presence of predators, presence of other competing mosquito species, vegetation coverage and intervention, water temperature, electrical conductivity, pH, dissolved oxygen, salinity, total dissolved solution and turbidity are among the physicochemical factors that affect the density and distribution of mosquito larvae [9, 10]. Similar studies in India, Benin and Iran indicated the correlation between environmental factors and *Anopheles stephensi and other Anopheles spss* larval abundance [11-13]. But there are no studies that demonstrated the correlation between water physicochemical parameters and *Anopheles stephensi* larval abundance in Ethiopia.

Globally, LSM (larval source management) has a proven track record in malaria control and even elimination in some countries. Historical successes in Brazil, Egypt, and parts of Asia relied heavily on environmental management [14]. More recent studies show that larviciding using biological agents like *Bacillus thuringiensis israelensis* (Bti) can reduce larval and adult vector densities by up to 90% [15]. In Ethiopia, larval source management is assumed to be a supplementary strategy besides the principal vector control interventions : use of insecticide treated nets and indoor residual spraying but there is inadequate larval identification and response to guide adoption of LSM approaches [16].

In Ethiopia, pilot studies have shown promising results with significant reductions in larval density and adult vector population reduction through regular Bti and Sumilarv application [17, 18]. Similarly, a study in Eastern part of the country on larval habitat suggested that LSM could be effective for focused control of *An.stephensi* [19]. In addition, LSM integrated with LLINs and IRS resulted in a notable reduction in malaria incidence in Tolay area of oromia region and in northern Cot^e Idvore’ ([20, 21]. In contrary, Ayana et al, 2025 reported that there was an increased malaria cases irrespective of LSM implementation and need to investigate factors that might affect the success of the intervention [22]. Therefore, this study was conducted to assess the physicochemical characteristics of larval habitats and relation with the ecology, season, larval density and distribution of larvae across the urban, peri-urban and rural areas of Dire Dawa.

## Methods

### Study area

**Figure 1:**
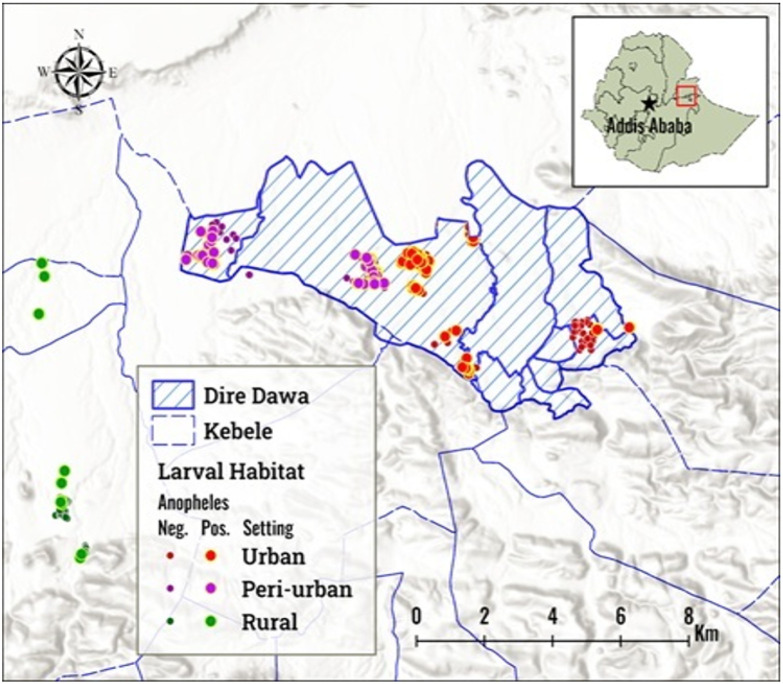
Map of the study area in Dire Dawa City Administration, 2024. This map was created with ArcGIS Pro 3.5 by Esri. (www.esri.com).

The study was conducted in three ecological settings, of Dire Dawa City administration (515 Km East of Addis Ababa) which included urban, per-urban and rural settings. The urban setting considered for this study were Dire Dawa University, GTZ and Mermersa. The peri-urban sites were those around the industrial zone including villages of Melka-Jebdu and Jerba. The rural study sites were Aseliso, Hulahulul and Boren. The rural sites were agricultural sites with grass land and scanty vegetation coverage. (Fig. 1)[23]. During the study, larval source management was implemented jointly by the President’s Malaria Initiative (PMI) and the Ministry of Health of Ethiopia in the urban and peri-urban sites [24].

The study area has seasonal malaria transmission, with *Plasmodium falciparum* and *P. vivax* the main parasite species,and the vectors include *Anopheles arabiensis* and *An. phareonsis.* Recent studies reported the occurrence of *An. stephensi* as an additional vector [25, 26].

### Immature mosquito collections and larval habitat characterization

Larval surveys were conducted following standard WHO protocol and mosquito immatures were collected using larval dipper of 350ml capacity [27, 28]. Twenty dips were taken from each habitat by dipping at different sides of the habitat. Each larval instar and pupae were counted and recorded. Data were entered monthly using open data kit (ODK) immediately synced to the International Centers of Excellence for Malaria Research (ICEMR) database server to avoid data loss. Geographic coordinates, ecological setting (urban, peri-urban and rural), habitat type (Artificial and Natural) and picture of the habitat on spot, source of water (tap ,rain, or river), distance from household, land use (surrounding environment of the habitat), shade status/degree of exposure to sunlight, water flow, substrate type, distance from brick making, distance from nearest resting habitat and frequency of utilization, presence of canopy cover, presence of intervention, type of intervention and presence of predators were included among others. Mosquito immatures were classified as Anopheline, Aedine and Culicine based on morphological observations. The species composition of *Anopheles* mosquitoes were determined from adults reared from larvae or pupae.

### Molecular species identification

The collected adult *Anopheles* were identified first to species using the morphological keys of Coetzee *et al*. 2020 and Glich *et al*. 1995 [29, 30]. Those identified as *An. gamabiae* s.l were further re-identified using species specific PCR. Real time PCR was also employed to confirm *An. stephensi* at the Genomics Laboratory of Jimma University Tropical Infectious and Diseases research center (TIDRC).

### DNA extraction and amplification

DNA extraction was done using both the Chelex and Extracta protocols [31]. PCR amplification was carried out according to the methods of Scott *et al*. [32] using species-specific primers for *An. arabiensis* (AR: 5'-AAG TGT CCT TCT CCA TCC TA-3') and *An. amharicus*, formerly *Anopheles quadriannulatus* B (QD: 5'-CAG ACC AAG AGA GAT GGT TAG TAT-3')[33]. *Anopheles gambiae* (GA: 5'-CTG GTT TGG TCGGCA CGT TT-3') and a universal primer (UN: 5'-GTGTGC CCC TTC CTC GATGT-3'). Then the amplicon was loaded on a 2% agarose gel stained with ethidium bromide and run for gel electrophoresis. *Anopheles arabiensis* from the Sekoru insectary colony and *An. amharicus* specimens from Arjo area were used as positive controls [34].

### Physico-chemical analysis of larval habitats

Physicochemical parameters of each habitat were measured using HANNA (H198184) instrument of multiparameters (Multiparameter Meter, Hanna Instruments Inc., Woonsocket, Rhode Island, 02895, USA) after proper calibration of the instrument with the manufacturer’s probe solutions that was provided together with multiparameter. Measurements of acidity and alkalinity (pH/mV), oxidative reduction potential (ORP), electrical conductivity (EC), total dissolved solids (TDS), resistivity, salinity, seawater, dissolved oxygen (DO), atmospheric pressure and temperature were taken from 102 larval habitats.

### Statistical Analysis

Data were entered in to SPSS version 27 and descriptive analysis was used to compare frequency, distribution, and magnitude of *Anopheles* larvae in the urban, peri-urban and rural interfaces. Multiple regression analysis was used to compare the effect of physicochemical variables of habitats on the *Anopheles* larva presence / absence, and larval density. Principal component analysis (PCA) was performed and Spearman’s correlation coefficient were used to dictate the correlation among water physicochemical parameters and larval presence and density. Cross tabs, Pearson’s Chi squared and Fisher’s exact tests were used to compare the association of presence/absence of predators, and presence/absence of intervention on larval presence and density. Larval density was calculated as number of larvae/pupae per 20 dips per habitat. Habitat index, container Index, and breteau Index were used to compare the larval density and abundance in each of the three ecological settings. Standard mathematical formulae [35] were used to calculate the larval indices as follows: 1) House Index (HI) was calculated by dividing the number of houses infested to the total number of houses inspected and multiplied by 100.

2) Container Index (CI) was calculated by dividing the number of containers positive for anopheles larvae to the total number of containers inspected and multiplied by 100. 3)Breteau Index (BI) was calculated by dividing the number of positive containers to total number of houses and multiplied by 100.

## Results

### Larval habitat abundance and indices

**Figure 2:**
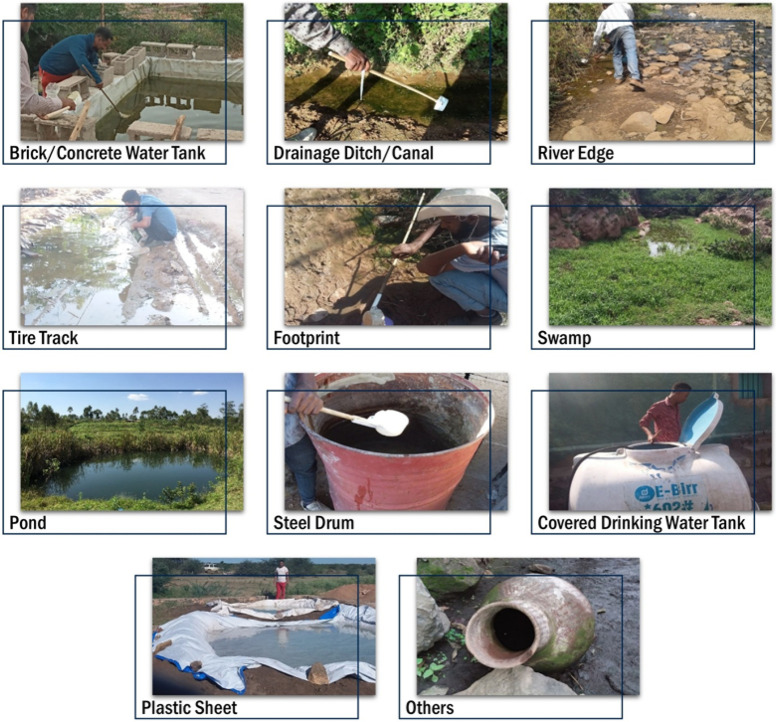
Types of larval habitats in Dire Dawa City Administration

A total of 909 habitats were surveyed in Dire Dawa, of which, 857 (94.3%) were artificial breeding habitats such as bricks/concrete water tankers, plastic sheets, plastic water tankers (barrels), steel drums, canal ditches and tire tracks while the other were natural habitats such as river edges, animal hoof prints, ponds and swamps (Table 1)(Fig. 2).

In Dire Dawa, uncovered water tanker/Cemented cistern or brick were the predominant habitat type 520/909 (57.2%) and followed by plastic sheet 221/909 (24.3%). *Anopheles* larvae were found in 297 (32.7%) of the houses surveyed and in 400(43.7%) of containers inspected in Dire Dawa (Table 1 and Table 2).

As depicted below in Table 2, about 42, 34%, and 24% of larva habitats surveyed were from urban, peri-urban and rural settings respectively.

Nine hundred-fifteen containers were found in houses surveyed with high number of containers 350(38.3%) in urban, 316 (34.5%) in peri-urban and 249 (27.2%) in rural sites respectively.

A total of 23,526 larvae and 1,808 pupae of *Anopheles* mosquitoes were collected from 909 habitats in urban, peri-uban, and rural areas of Dire Dawa. There was a difference in monthly larval density at each ecology and habitat type.

Paired-samples t-tests indicated that there is a significant difference in the density of *Anopheles* larvae across months (t=−3.27, n = 34, *p* = .003) with a mean difference of − 6.98 larvae per dip (95% CI: −11.32 to − 2.63) and a medium effect size (Cohen’s d = 0.55), indicating a moderate seasonal influence on larval abundance. Similarly, the paired-samples t-test showed that larval density differed significantly between ecological categories ( t = −5.37, n = 34, *p* < .001) with a mean difference of − 11.52 larvae per dip (95% CI: −15.88 to − 7.16) and a large effect size (Cohen’s d = 0.91). A stronger effect was observed across ecological settings, which suggests that ecological characteristics exert a greater influence on *Anopheles* larval density than seasonal variation alone.

**Figure 3:**
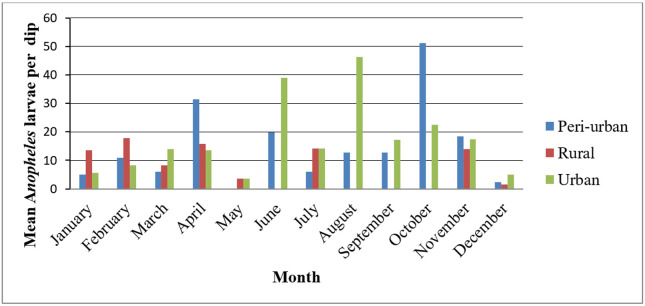
Mean *Anopheles* larval density by month and ecological setting in in Diredawa, Ethiopia (2024)

In urban sites, where larval density was estimated to be constant throughout the year, the monthly *Anopheles* larval density was high in October and lowest in May in urban settings. In peri-urban sites, *Anopheles* larval density was highest in October and absent in May. In rural sites, larval density was peak in February and absent from June to October (Fig. 3), showing that there was a significance difference between months and ecology, where some ecological settings were absent in some months (*t* = 7.38, n = 34, *p* < 0.001), suggesting that temporal and ecological factors together influence larval dynamics.

The mean larval density were highest in peri-urban site in artificial uncovered water tanker (51 larvae per dip), followed by natural ditch in urban site (46 larvae per dips), and lowest in rural and urban plastic sheet habitats, and lowest in plastic sheets in Urban sites (0 per dip/habitat).

Habitat type differs significantly across the season (t = 6.36, n = 77, *p* < 0.001) indicating seasonal variation affect habitat type. Anopheles larvae abundance and density were significantly different in different types of habitats (t = −4.8, n = 77, *p* < 0.001). There is a significant association between ecological settings (urban, peri-urban, and rural) and habitat types (t=−6.027, n = 77, *p* < 0.001). Seasonal variations, specifically dry and long rain seasons, strongly affect larval abundance and density (t = −4.95, n = 77, *p* < 0.001).

**Figure 4:**
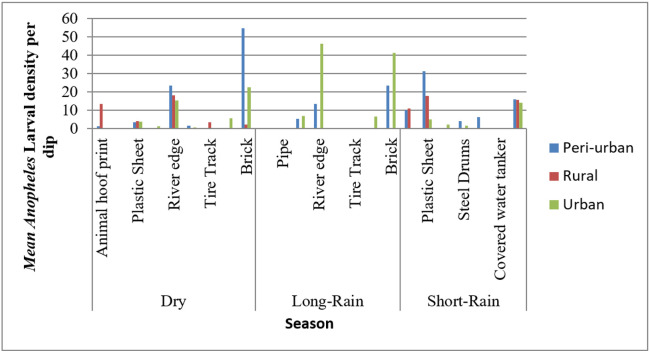
Mean *Anopheles* larval density by season, habitat type and ecological setting in Dire Dawa City Administration, Ethiopia (2024)

As construction of houses and production of bricks are common in urban and peri-urban areas of Dire Dawa, *Anopheles* larvae population density was high throughout the year in the artificial water storages, regardless of the seasonal variation in the urban and peri-urban ecologies. But in rural areas, *Anopheles* larval density increased following the short rain season (Fig. 4).

Proximity of larval habitats to houses 80% (732 /909) were located in less than 50 meters from nearby living houses, 65% (589/909) of the habitats were at 0–50 meters away from brick making houses. Additionally, 56% (509/909) of the habitats surveyed were surrounded by construction site or land-use for brick making. Half of the total habitats substrate were made of concrete with 35% (320/909) covered with plastic sheet or lining and about 11% of the habitat substrate was mud. The results of logistic regression analysis indicated that man-made/artificial habitat and grass land of surrounding were significant predictors for the presence of *Anopheles* larvae/pupae (Table 3).

**Figure 5:**
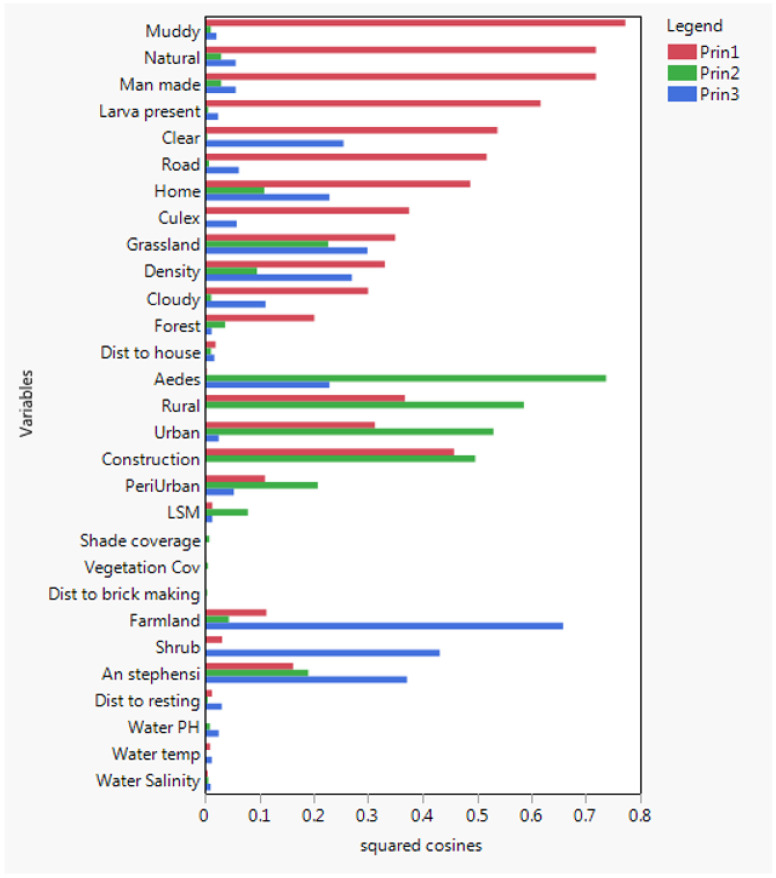
Variables grouped by PCA, Variables grouped together means they are correlated. PCA 1 (red bars) includes *Anopheles* and *Culex* larval presence, PCA 2 (green bars) includes *Aedes* larval density, PCA 3 (blue bars) includes *An. stephensi* presence

Both natural and man-made habitats were having same correlation with *Anopheles* and *Culex* larval presence and *Aedes* larval density. But rural sites were highly correlated with *Aedes* larval density than urban and peri-urban and sites. Conversely, *An.stephensi* larval presence were correlated with urban and peri-urban than rural sites. Similarly, the result of the PCA indicated positive correlation between land use (surrounding environment) and larval presence, habitat with in Grass-land environment, farm land, and availability of shrubs and in or near household were correlated with presence of *Anopheles, Culicines and Aedeines* larvae (Fig. 5 and Fig. 7).

**Figure 6:**
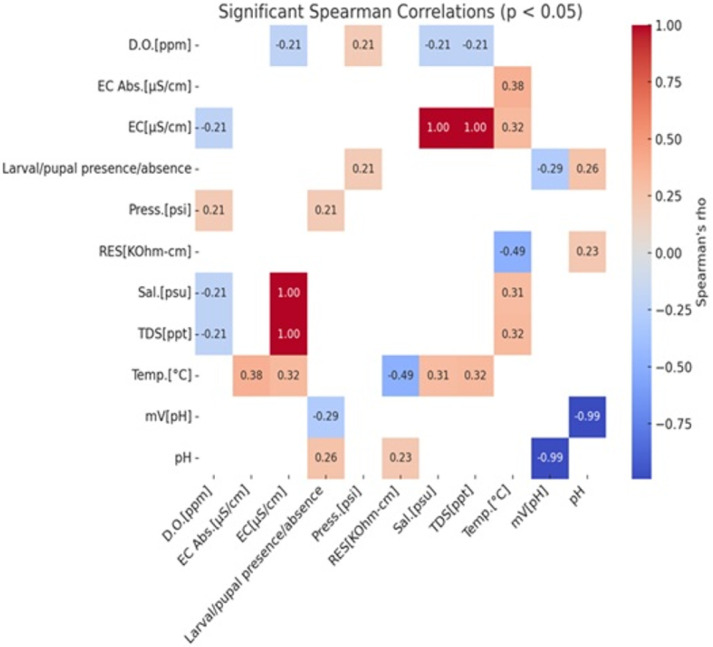
Results of Spearman’s correlation among predictors and occurrence of mosquito immatures.

The results of principal component analysis (PCA) indicated that Larva/pupa presence were positively correlated with pH (r = 0.264, *p* = 0.01) and water pressure (r = 0.21, *p* < 0.05). Similarly, there was a significant positive correlation with temperature and conductivity (EC) (r = 0.31, *p* = 0.02), Total dissolved solids (TDS) ((, r = 0.38, *p* = 0.02) and, Salinity (r = 0.38, *p* = 0.02)(Fig. 6).

EC, TDS and salinity were positively correlated (r = 1, *p* = 0.00). Also Dissolved oxygen (DO) and water pressure (psi) showed positive correlation (r = 0.211, *p* = 0.040). But, temperature and resistivity had negative correlation (r = − 0.494, *p* = 0.00), pH and mvpH also had negative correlation (r= −0.993, *p* = 0.00). In addition, there was no significant correlation between temperature and Dissolved oxygen, Dissolved oxygen and Salinity (*p* > 0.05). Oxydative reduction potential (ORP) was the variable that had no-significant correlation with the dependent variable (Fig. 6).

**Figure 7:**
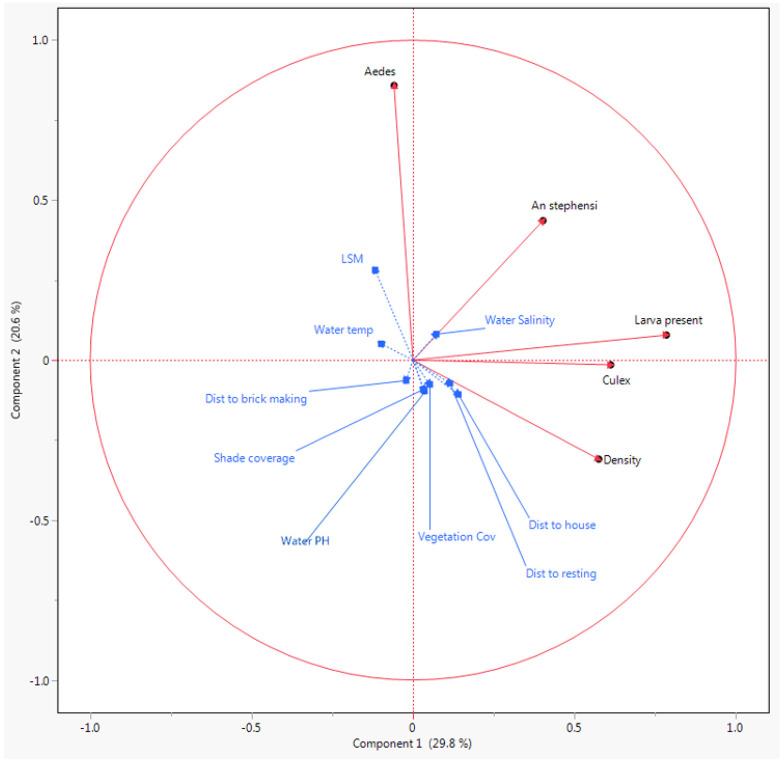
Correlation of Environmental and physicochemical parameters with presence and density of mosquito immatures.

The PCA result also showed that *Anopheles* larval/pupa density were positively correlated with shorter distance to living house and distance to resting places, availability of brick making, vegetation cover, shading, pH and salinity (Fig. 7).

The Pearson’s Chi squared test revealed a significance association between the predators and competitors presence on larval presence ( ^2^=16.3, df:1, *p* < 0.01). Larvae were found more often in locations where predators were present (66.7%) compared to locations without predators (31.5%). The result indicated that larva presence and predator presence are not independent, and predator and competitors presence is significantly associated with higher likelihood of larva presence.

But, the Pearson’s chi squared test indicated that there is no significance association between presence of intervention (larviciding) and larval presence ( ^2^ = 2.66, df = 2, *p* = 0.264), the presence of intervention doesn’t affect larval presence (*p* > 0.05).

As depicted in Fig. 5, Clear water had positive correlation with presence of *Anopheles stephensi*. Moreover, water salinity, water temperature and water pH were among the strong predictors of the presence of *An.stephensi* larvae (Fig. 7)

The analysis of water salinity revealed that the majority of samples (88.3%) fell within the valid or normal salinity range, indicating predominantly fresh water conditions across the study area. Only 11.7% of the total 873 samples exhibited measurable salinity above 0 ppt. Among these, slightly saline water (2–3 ppt) was the most frequent, accounting for 5.6% of the samples, followed by low salinity levels between 0–1 ppt (2.4%). Higher salinity categories were much less common, with only 1.8% of samples in the 4–5 ppt range and less than 1% exceeding 6 ppt. Extremely saline samples (> 30 ppt) represented just 0.3% of the total (Table 5).

The water physicochemical analysis indicated that water temperature of larval habitat ranged from 19°C to 34°C. The water temperature for 80.4% (82/102) of larval habitats was from 20–27°C (room temperature), 16.6% (17/102) of the larval habitats water temperature was from 28–34°C, and for the remaining 2.9% (3/102) of habitats, the water temperature recorded were 19°C.

The water pH values measured and categorized in to three as acidic water (pH value < 7), neutral (pH value = 7) and alkaline (pH > 7).The result indicated that 86.3% (88/102) of habitats were alkaline, 12.7% (13/102) were neutral and 0.98% (1/102) of habitats were acidic.

Five types of substrate were considered (Gravel, Sand, Mud, Concrete and Plastic cloth) and the result of the study showed that 50% (52/102) of the habitats substrate were made of concrete and 25% (26/102) of the habitat substrate were made of plastic cloths/container. Fisher’s exact test indicated that there is a statistical association between larval presence and substrate type ( ^2^=26.6, df = 6, *p* < 0.01).

### Species diversity and evenness

A total of 2,934 adult *Anopheles* mosquitoes were emerged from immatures from all sites and *An. stephensi* was the predominant species 75% (2194/2934) followed by *An.arabiensis* 22% (636 / 2934), *An. pharoensis* 1.7% (50/2934), *An. coustani* 1.3% (38/2934), *An.amharicus* 0.3% (9/2934) and *An. pretoriensis* 0.2% (7/2934), respectively (Table 5).

Further paired samples *t*-test revealed significant differences in species composition between habitat categories (man-made and natural). Abundance differed significantly between habitats (*t*= − 2.22, n = 13, *p* = 0.004), with man-made habitats having higher *Anopheles* abundance. Significant habitat-related differences were also observed in Shannon diversity (t = 10.03, n = 13, *p* < 0.001), species evenness (t = 5.70, n = 13, *p* < 0.001), and species richness (t = 5.7, n = 13, *p* < 0.001), indicating greater diversity, more even species distribution, and higher species richness in natural habitat category.

*An. stephensi*, *An.arabiensis* and *An.amharicus* found to share same artificial habitats more specifically plastic sheets (in urban, peri-urban and rural), and in natural habitats more specifically a river Butuji river found in Dire Dawa city.

#### Molecular identification of An.gambiae s.l and An.stephensi

Of total 645 *An.gambiae* s.l sub-samples that were tested using PCR, 98.6% (n = 636) were found to be *An.arabiensis*, and 1.4% (n = 9) were *An.amharicus*. Similarly, real time PCR (qPCR) was performed to confirm morphologically identified *An.stephensi* specimens from *An.gambiae* s.l, and accordingly 200 sub-samples of each *An.stephensi* and *An.arabiensis* were proved distinct.

## Discussion

Our study indicated that *An.stephensi* immature stages are abundant throughout the year regardless of the seasonal variation of rain, with peak larval densities in dry season in all the three ecological settings. Similar results were reported by Yared *et al*. 2023 [19] in Somali region, Jigjiga town, where larvae were present in dry season. This might be because both Dire Dawa city and Jigjiga town have similar in expansion of urbanization and construction which support the breeding of immature stages in dry season.

Moreover, larval habitats were found in less than 50m from living houses and brick making houses were located at distance ranging from 0-50m away from living houses. This finding has an important implication as far as supporting the life cycle of *Anopheles* and maximizing the probability of man vector contact. This finding could help to implement targeted larviciding, larval source management in habitats that harbor the immature stages throughout the year.

The other finding of our study is the co-existence of *An.stephensi* with *An.gambiae s.l*, sharing same both artificial and natural habitats and ecological settings. *An.stephensi* were known to be vector of malaria mainly in urban settings and in an artificial habitats [6, 11], but in our study *An.stephensi* were found in rural ecology in Dire Dawa, and in river edges of Legehare and Butuji rivers, the two known and permanent habitats in the city. This further remarks the expansion of *An.stephensi* to rural sites and adapting natural habitats to breed. Our finding coincides with a study done in South east Iran where *An.stephensi* were found in river edges with sandy substrates [12].

Unexpected result of our research in the study area is, the presence of *An.amharicus* which were reported in South Western, Central, Western, [33, 36] and most recently in North west part of the country, here in the Eastern part of Ethiopia ,we report the existence of *An.amharicus* in urban,peri-urban and rural ecologies of Dire Dawa for first time. Additionally, *An.coustani*, *An.pretoriensis and An.phareonsis* were found in the study area.

This finding recommends further investigation on the role of *An.amharicus* and the other vectors in malaria transmission in Dire Dawa and their general bionomics including its susceptibility to current insecticides in use are important.

Water physicochemical analysis indicted that larval distribution and abundance was not significantly different as the measures of the water physicochemical vary. Also a change in Ecology, season, habitat category doesn’t indicate a difference on presence and absence of larvae and density of larvae in man-made habitats. Our study align with the study conducted in Northern Iran which indicated no significance difference in physicochemical characteristics and larval density [37].

In contrast to our study, that indicated the analysis of water salinity revealed that the majority of samples (88.3%) fell within the valid or normal salinity range and the 86% fell in alkaline pH, indicating predominantly fresh water conditions across the study area, studies on correlation of *Anopheles* larval density with physicochemical characteristics in Benin and South east Iran indicated that there was high positive correlation of larval density with temperature [13], dissolved oxygen and salinity [12, 13].This difference might be because of the difference in the source of water in urban areas of Benin and South east Iran from our study area where almost 98% of source of water for larval habitats were ground water and chemical characters were in similar ranges.

In agreement with a study conducted by Mereta et al. 2013 [9], our study found that there was wide spread distribution of *Anopheles* larvae in small man-made aquatic habitats but in contrast to Mereta eta al. 2013 finding, in our study, *Anopheles* larva was abundant in both natural and artificial habitats.

Year-round presence of larvae in artificial habitats indicated larval density, distribution and abundance were not affected by season as far as water supply continued for the purpose of mainly for construction and brick making. Expansion of *An.stephensi* to rural sites could be due to the rapid urbanization and industrial expansion which is accompanied with construction of water storages. The *An.stephensi* samples found in rural sites (40km away from Dire Dawa city) in Aseliso was in plastic sheet habitat type implicated that where ever the artificial habitats are used they are suitable for the exotic species to breed in. This study indicated the strong association of some ecological settings with the density and abundance of *An.stephensi* in urban and peri-urban than rural sites this could be due to availability of enormous construction and brick manufacturing in urban and peri-urban settings.

## Conclusion

*An.stephensi* was the predominant species found in the study area, followed by *An.arabiensis, An.amharicu, An.pharoensis, An.coustani and An.pretoriensis*. Uncovered water tankers were the most efficient habitat types in urban and peri-urban sites followed by plastic sheet habitat types while hoof prints and river basins under natural habitats were the most efficient habitat types in rural and urban sites respectively. *An. stephensi* was found in rivers of urban site and plastic sheets of rural sites co-existing with *An.arabiensis* in same sites. Both shared same ecological settings and habitat types. *An.amharicus* was found to breed in artificial habitats of the three ecological settings.

We report here the existence of *An.amahricus* for the first time in the study area, Eastern part of Ethiopia, expansion of *An.stephensi* in rural areas, breeding in natural habitats, and co-existing with *An gambiae s.l*.

Availability of brick making, shorter distance from living houses being in urban ecology were directly correlated with larval density. The result of our study revealed that there is significant correlation of ecological and seasonal variation in the density of *Anopheles* larvae. Habitat abundance and positivity of uncovered water tankers in urban and pre-urban sites could indicate for feasibility and proper implementation of larval source management.

### Limitation of the study

The physicochemical tests were performed in a cross-sectional way but we believe to be done during main rain season to observe if seasonal variation affects the water chemistry.

## Figures and Tables

**Table 1:** Larval habitat abundance, proportion and larval presence positivity in urban, peri-urban and rural sites in Dire Dawa City Administration (2024)

Habitat type		n*(%)	positive for Anopheles(%)
	Brick concrete water tanker	520 (57.2)	187(36.0)
	Drainage ditch/canal	10 (1.1)	2 (20.0)
	River edge	25 (2.8)	14 (56.0)
	Tire Tracks	6 (0.7)	4 (66.6)
	Foot Print	11 (1.2)	9 (81.8)
	Swamp	6 (0.6)	4 (66.7)
	Pond	10 (1.2)	4 (40.0)
	Steel Drum	25 (2.8)	5 (20.0)
	covered drinking water tanker/Birca	60 (6.6)	8 (13.3)
	Plastic sheet	221 (24.3)	58 (26.2)
	Other (small water containers, pipe, foundation, septic tank)	15 (1.7)	2 (13.3)
	Total	909 (100)	297 (33)

**Table 2:** Larval indices and the distribution of habitats in urban, peri-urban and rural ecologies in Dire Dawa City Administration, Ethiopia

Site	Houses surveyed n (%)	# positive houses n (%)HI*	#containers n (%)	#Positive containers CI* n (%)	Breteau Index BI* n (%)
Urban	385 (42.0)	133 (34.5)	350 (38.3)	175 (50)	175 (45.5)
Peri-urban	307 (34.0)	107 (34.8)	316 (34.5)	130 (41.1)	130 (42.3)
Rural	217 (24.0)	57 (26.3)	249 (27.2)	95 (38.2)	95 (43.8)
Total	909 (100)	297 (32.7)	915 (100)	400 (43.7)	400 (44)

N = number, *HI-House index, CI: Container index and BI: Breteau index

**Table 3:** Logistic regression analysis for the assessment of predictors of mosquito larval occurrence in urban, peri-urban and rural areas of Dire Dawa City Administration, Ethiopia

Variable	Estimate	ChiSquare	Prob > ChiSq	Lower 95%	Upper 95%
Eco setting[peri-urban]	0.249	1.34	0.2469	−0.172	0.675
Eco setting[rural]	−0.251	1.03	0.3103	−0.747	0.228
Habitat category[man made]	2.222	5.76	0.0164	0.823	4.805
Landuse2[construction]	0.199	0.19	0.6608	−0.638	1.148
Landuse2[farmland]	0.407	0.7	0.4023	−0.534	1.386
Landuse2[grassland]	1.504	4.45	0.0349	0.165	3.019
Landuse2[house]	−0.588	1.05	0.3058	−1.717	0.557
Landuse2[road]	−1.943	1.28	0.2587	−5.645	1.139
Vegetation Cover	−0.008	0.23	0.6321	−0.041	0.027
Shade coverage	0.001	0.01	0.942	−0.018	0.018
LSM	−0.525	0.88	0.3494	−1.693	0.540
Distance to house	−0.004	2.26	0.1325	−0.010	0.001
Distance to resting	0.000	0	0.988	−0.005	0.005
Distance to brick making	−0.003	1.69	0.1932	−0.009	0.001
Habitat length	−0.031	0.86	0.3531	−0.107	0.032

**Table 4:** Results of water salinity measurements of larval habitats in Dire Dawa City Administration, Ethiopia

Salinity (ppt)	N (%)	Category
**0–1**	21 (2.4)	Very low salinity (freshwater).
**2–3**	49 (5.6)	Slightly saline (brackish tendency).
**4–5**	16 (18)	Moderately saline.
**6–7**	6 (0.7)	Noticeably saline.
**8–9**	2 (0.2)	Highly saline.
**10–14**	5 (0.6)	Very saline—approaching seawater levels.
**> 30**	3 (0.3)	Extremely saline (seawater or hyper-saline).

**Table 5:** Anopheles mosquito species diversity, richness and evenness by habitat type in Dire Dawa City Administration, Ethiopia

Habitat	Species	Abundance	Proportion (pi)	Lnpi	pi*Lnpi	Shannon diversity index (H')	Species eveness (J): H/LnS	Species richness (S)
Artificial	*An.stephensi*	1873	0.91	−0.1	−1.	0.1	0.3	3
	*An.arabiensis*	185	0.09	−2.4	−0.2	0.22		
	*An.amharicus*	8	0.00	−5.6	−0.1	0.02		
	*An.pretoriensis*	0	0	0	0	0		
	*An coustani*	0	0	0	0	0		
	*An.pharoensis*	0	0	0	0	0		
*Total*		2066	1	0	0	0.33		
Natural	*An.stephensi*	321	0.37	−1	−0.4	0.4	0.6	6
	*An.arabiensis*	451	0.52	−1	−0.3	0.3		
	*An.amharicus*	1	0.00	−7	−0.01	0.01		
	*An.pretoriensis*	7	0.01	−5	−0.04	0.04		
	*An.coustani*	38	0.04	−3	−0.14	0.14		
	*An.pharoensis*	50	0.06	−3	−0.16	0.16		
*Total*		868	1	0	0	1.06		
